# PW01-003 – Frequency of MEFV mutations in Turkish population

**DOI:** 10.1186/1546-0096-11-S1-A56

**Published:** 2013-11-08

**Authors:** F Yalcinkaya, A Duzova, S Gonen, B Ozcakar, E Baskin, O Ozkaya, O Soylemezoglu

**Affiliations:** 1 Ankara University, Turkey; 2 Hacettepe University, Turkey; 3 Gazi University, Turkey; 4 Baskent University, Ankara, Turkey; 5 Ondokuz Mayis University, Samsun, Turkey

## Introduction

Data on the epidemiology of familial Mediterranean fever (FMF) and the prevalence of disease causing mutations among different ethnic groups and geographical regions around the world are insufficient. The prevalence of mutations that account for FMF in Turkey has been defined in the past by determining the frequency of MEFV mutations in affected individuals or in hospital-based controls. This study is a population-based study and is, therefore, different from previous patient-based studies.

## Objectives

To investigate the prevalence and distribution of MEFV mutations in Turkish population with a nationwide population-based study.

## Methods

Subjects were included from 12 statistical regions according to the EuroStat NUTS level 2. The distribution of the study population was parallel with the general Turkish population according to gender, residence, and geographical regions. To date a total of 388 unrelated healthy Turkish participants (M/F:189/199; age:5.3-79.75 years) were tested for 10 mutations in the *MEFV* gene: p.A761H, p.A744S, p.V726A, p.K695R, p.M694V, p.M694I, p.M680I (G®A) in exon 10, p.F479L in exon 5, p.P369S in exon 3, and p.E148Q in exon 2, using pyrosequencing technique.

## Results

Our results showed that 62 of 388 participants (16.0%) (95% CI:12.5-20.0) were carriers of MEFV mutations. Seven individuals were compound heterozygous, two homozygous and 53 were heterozygous for the mutations. Mutation frequency was 9.2% (95% CI: 7.22-11.4). The most common mutations in the Turkish general population were p.E148Q, p.M694V and p.P369S and the frequencies were 3.6% (95% CI: 2.4-5.2), 2.6% (95% CI: 1.6-4.0) and 1.0% (95% CI: 0.5-2.0), respectively.

## Conclusion

Our study shows a high frequency of carriers and independently confirms that M694V is the second most common mutation in the healthy Turkish population. If all the patients who had two most common mutations present the disease is anticipated the results of our study show that the disease is more common than predicted and one can speculate that patients with mild clinical findings might exist.

## Disclosure of interest

None declared

